# Supplementary material for: ILF3 contributes to the establishment of the antiviral type I interferon program
Source: Nucleic Acids Res. 2019 Nov 8;48(1):116–29. doi: 10.1093/nar/gkz1060 (PMC7145544; doi:10.1093/nar/gkz1060)
Supplement: gkz1060_Supplemental_Files [file gkz1060_supplemental_files.zip › Watson_suppl material.pdf]

Supplementary Figure 1

A

Total RNA-sequencing

Total-RNA seq (rRNA-depleted)	Total number of reads	% mapped to GencodeV24 (Salmon)	% mapped to hg38 (STAR)
siMock 1	33,511,350	60.20%	87.33%
siMock + p(IC) 1	33,259,656	49.49%	88.47%
siILF3 1	29,509,756	64.34%	87.19%
siILF3 + p(IC) 1	42,882,373	51.88%	88.6%
siMock 2	30,307,012	64.10%	86.29%
siMock + p(IC) 2	35,991,928	49.84%	87.93%
siILF3 2	35,906,394	62.96%	86.04%
siILF3 + p(IC) 2	36,874,925	52.52%	87.71%
siMock 3	31,616,855	67.11%	86.96%
siMock + p(IC) 3	27,843,328	47.11%	87.48%
siILF3 3	33,429,895	63.71%	85.86%
siILF3 + p(IC) 3	34,242,526	54.73%	86.87%

Total number of reads obtained for each of the replicates and conditions from total RNA sequencing libraries. Reads were mapped using Salmon to GencodeV24 for downstream analyses. Alternatively, reads were mapped to the hg38 human genome using STAR. The % of mapped reads using SALMON is artificially low due to the lack of annotation of very abundant non-coding RNAs in this database, such as long non-coding RNAs, rRNAs, tRNA and snRNAs.

B

KEGG analyses FDR<0.05

UPREGULATED siMock p(I:C) vs siMock

Cytokine signaling in immune system	4.27E-11
Cytokine - cytokine receptor interaction	2.87E-08
Interferon alpha/beta signaling	3.14E-08
Gene transcription pathway	1.93E-07
RNA polymerase II transcription	5.53E-06

UPREGULATED siILF3 p(I:C) vs siMock

Cytokine signaling in immune system	4.62E-15
Cytokine - cytokine receptor interaction	3.81E-11
Interferon alpha/beta signaling	5.23E-08
Signaling by interleukins	9.07E-07
Immune system	9.18E-07

DOWNREGULATED siMock p(I:C) vs siMock

Translation	1.91E-74
Metabolism of proteins	1.11E-46
Metabolism	3.40E-43
Metabolism of RNA	3.08E-35
SRP-dep cotranslational protein targeting to membrane	6.55E-29

DOWNREGULATED siILF3 p(I:C) vs siMock

Translation	7.43E-65
Metabolism	4.85E-50
Metabolism of proteins	3.02E-40
Eukaryotic translation initiation	6.22E-26
Cap-dependent translation initiation	6.22E-26

KEGG and reactome analyses of differentially upregulated (top left) and downregulated (bottom left) genes during the antiviral response. Similar KEGG reactome categories are obtained during the antiviral response in the absence of ILF3, for both upregulated (top right) and downregulated (bottom right) groups. Only the top 5 categories are included.

Supplementary Figure 2

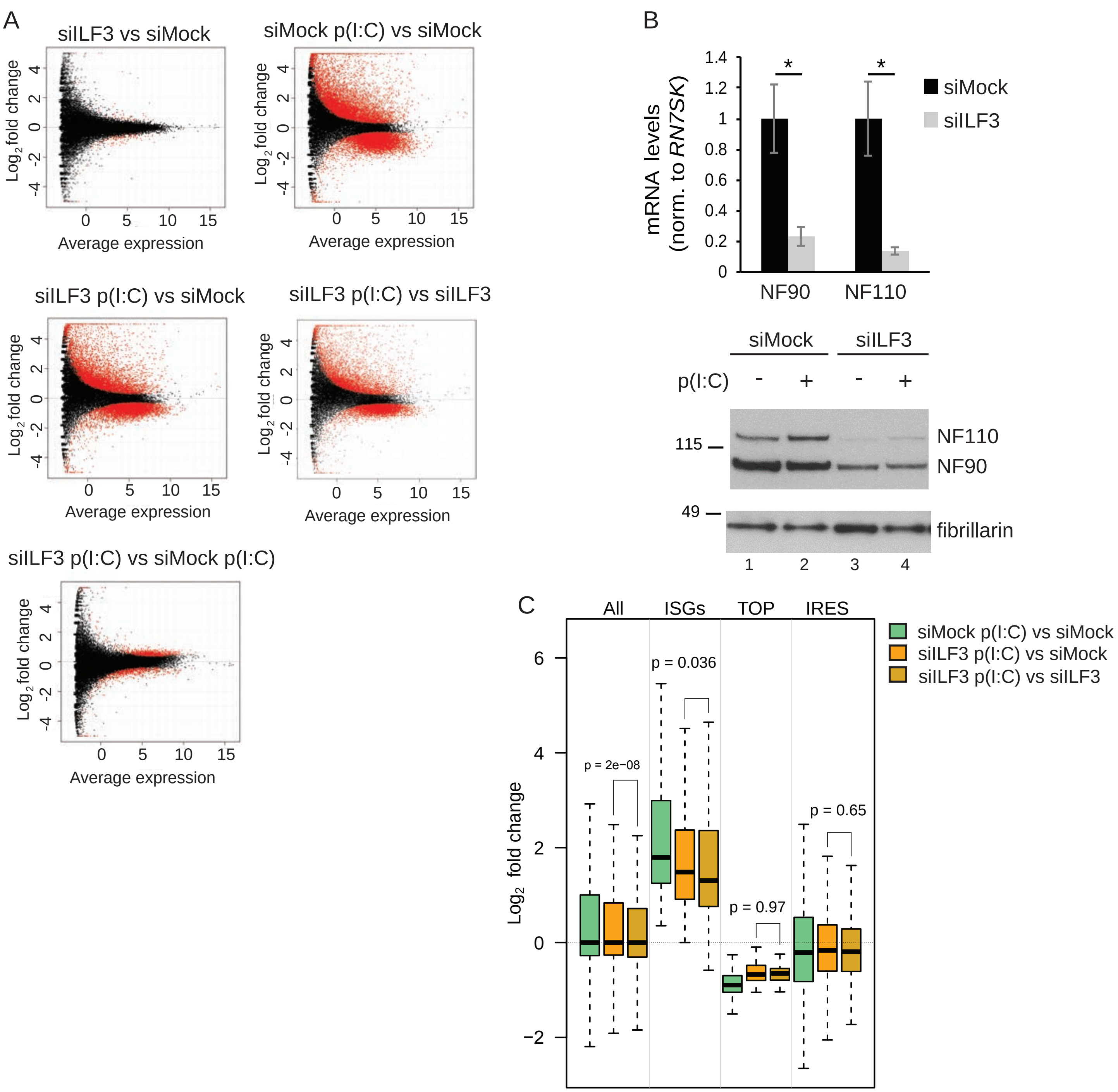

Supplementary Figure 2

(A) Differentially expressed genes in red (FDR q-value <0.05) during (**1**) ILF3 depletion in homeostasis (siILF3 vs siMock), siMock represents cells transfected with non-targeting siRNAs (**2**) during the antiviral response in the presence of ILF3 (siMock p(I:C) vs siMock) (**3**) during the antiviral response in the absence of ILF3 (siILF3 p(I:C) vs siMock and siILF3 p(I:C) vs siILF3) and (**4**) ILF3-dependent genes during the antiviral response (siILF3 p(I:C) vs siMock p(I:C)).

(B) (top) HeLa cells were depleted for 72 hours with siRNAs against ILF3 targeting both alternatively spliced isoforms produced by *ILF3* gene, NF90 and NF110. As a control, cells were transfected with non-targeting siRNAs (siMock). Isoform-specific primers were used for amplification of NF90 and NF110 isoforms to assess their relative mRNA expression levels upon ILF3 depletion. Data show the average n=3 +/- sd, (*) p-value <0.5 by Student's T-test (**Bottom**) Representative western blot analyses of NF90 and NF110 protein levels upon depletion with siRNAs against ILF3 (lanes 3 and 4), compared to mock siRNA transfected cells (lanes 1 and 2, siMock), in the presence (lanes 2 and 4) or absence (lanes 1 and 3) of antiviral response by transfection with the dsRNA analogue, p(I:C)

(C) Box-plot analyses of differential gene expression for: all genes, significantly induced ISGs, TOP and IRES mRNAs during the antiviral response(siMock p(I:C) vs siMock, as shown in Figure 1C) and in the absence of ILF3 during the antiviral response (siILF3 p(I:C) vs siMock and siILF3 (pI:C) vs siILF3), p-val by Mann-Whitney U test

Supplementary Figure 3

A

Polysome RNA-sequencing

Polysome-RNA seq	Total number of reads	% mapped to GencodeV24 (Salmon)
siMock 1	22,725,910	86.70%
siMock + p(IC) 1	22,262,276	82.43%
siILF3 1	22,129,182	93.28%
siILF3 + p(IC) 1	24,227,674	89.37%
siMock 2	22,806,334	91.56%
siMock + p(IC) 2	28,188,833	86.73%
siILF3 2	30,548,668	92.33%
siILF3 + p(IC) 2	22,863,763	90.14%
siMock 3	22,574,875	81.85%
siMock + p(IC) 3	21,690,479	81.38%
siILF3 3	23,930,254	91.42%
siILF3 + p(IC) 3	25,064,870	90,20%

Total number of reads obtained for each of the replicates and conditions from polysomal associated RNA sequencing libraries (polyA+ selection). Reads were mapped using Salmon to GencodeV24 for downstream analyses.

B

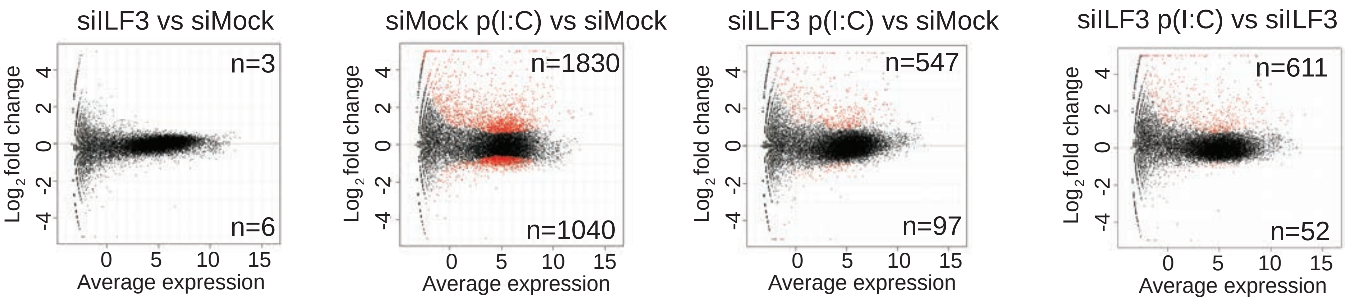

Differentially polysomal-associated protein-coding genes in red (FDR, q-value,0.05) during (1) ILF3 depletion in homeostasis (siILF3 vs siMock), (2) during the antiviral response (siMock p(I:C) vs. siMock) and (3) during the antiviral response in the absence of ILF3 (siILF3 p(I:C) vs. siMock and siILF3 p(I:C) vs. siILF3) (n=number of significant genes)

Supplementary Figure 4

A

KEGG and Reactome analyses

UPREGULATED
siMock p(I:C) vs siMock

n=1830

Cytokine Signaling in Immune system	3.67E-32
Signaling by Interleukins	1.26E-17
Cytokine-cytokine receptor interaction	2.34E-12
Senescence-Associated Secretory Phenotype (SASP)	2.99E-12
Systemic lupus erythematosus	6.50E-12

UPREGULATED
siILF3 p(I:C) vs siMock

n=547

Cytokine Signaling in Immune system	1.68E-34
Senescence-Associated Secretory Phenotype (SASP)	2.66E-20
Systemic lupus erythematosus	2.79E-20
Signaling by Interleukins	1.19E-19
HDACs deacetylate histones	4.00E-19

KEGG reactome analyses of genes more associated associated to polysomes during the antiviral response (left), and during the antiviral response in the absence of ILF3 (right). Only the top 5 most significant categories are shown.

B

KEGG and Reactome analyses of high expression group (polysome associated)

n=926

Translation	2.53E-65
SRP-dependent cotranslational protein targeting to membrane	1.36E-49
Ribosome	7.85E-43
Regulation of expression of SLITs and ROBOs	3.04E-41
Peptide chain elongation	4.16E-41

KEGG reactome analyses of highly enriched genes in heavy polysomal fractions (average expression logCPM > 5 pval<0.05)

C

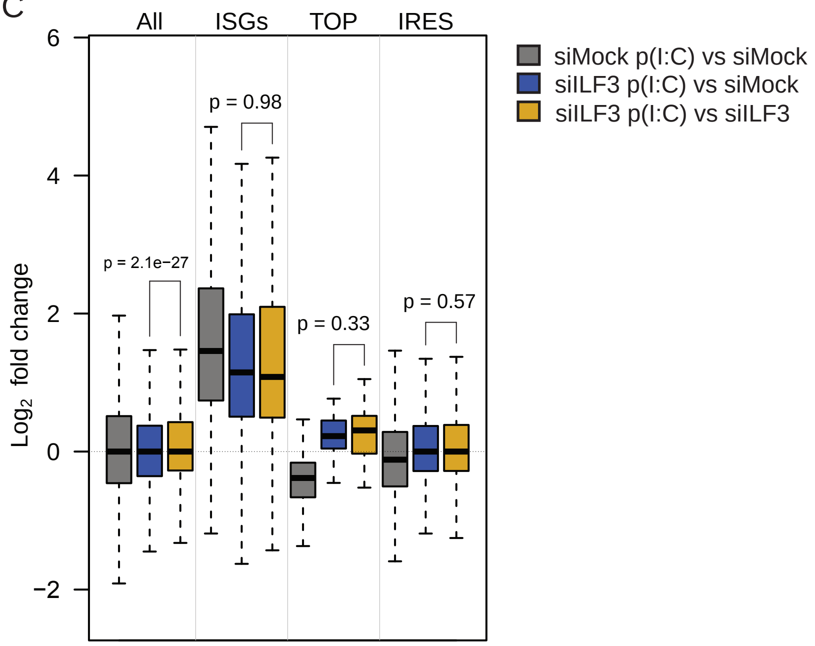

Box-plot of polysomal associated-mRNAs. Differential gene expression analyses were performed for all genes, significantly induced ISGs, TOP and IRES mRNAs during the antiviral response (siMock p(I:C) vs siMock, as shown in Figure 4B) and in the absence of ILF3 during the antiviral response (siILF3 p(I:C) vs siMock and siILF3 (pI:C) vs siILF3), p-val by Mann-Whitney U test

Supplementary Figure 5

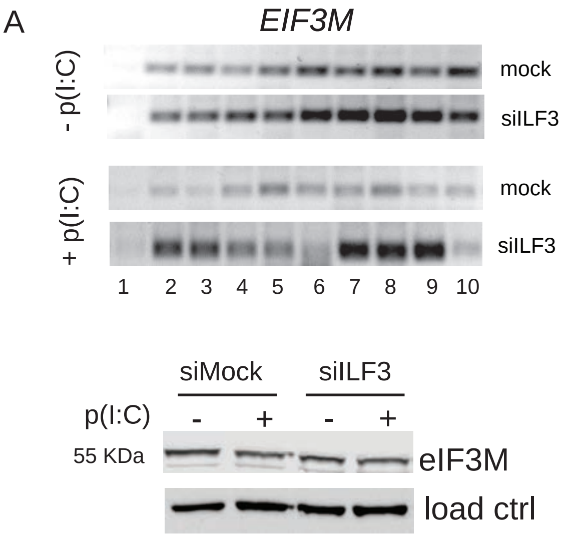

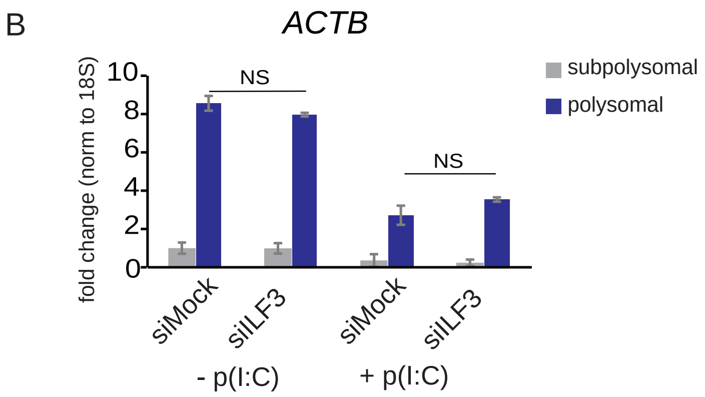

(A) (**top**) RT-PCR analyses of *EIF3M* co-sedimentation in each of the polysomal fractions collected as in Figure 3D (**bottom**) Western blot analyses of eIF3M protein levels in dsRNA-activated HeLa cells in the presence (siMock, lane 2) and absence of ILF3 (siILF3, lane 4). Fibrillarin serves as a loading control.

(B) qRT-PCR analyses of *ACTB* mRNA enrichment in subpolysomal (grey) and polysomal (blue) pooled fractions in siMock or siILF3 depleted HeLa cells +/- p(I:C). Data show the average (n=2) +/- s.e.m, normalised to 18S rRNA and relative to subpolysomal levels in mock, n.s. non-significant by two-way ANOVA followed by Tukey's multiple comparison test.

Supplementary Figure 6

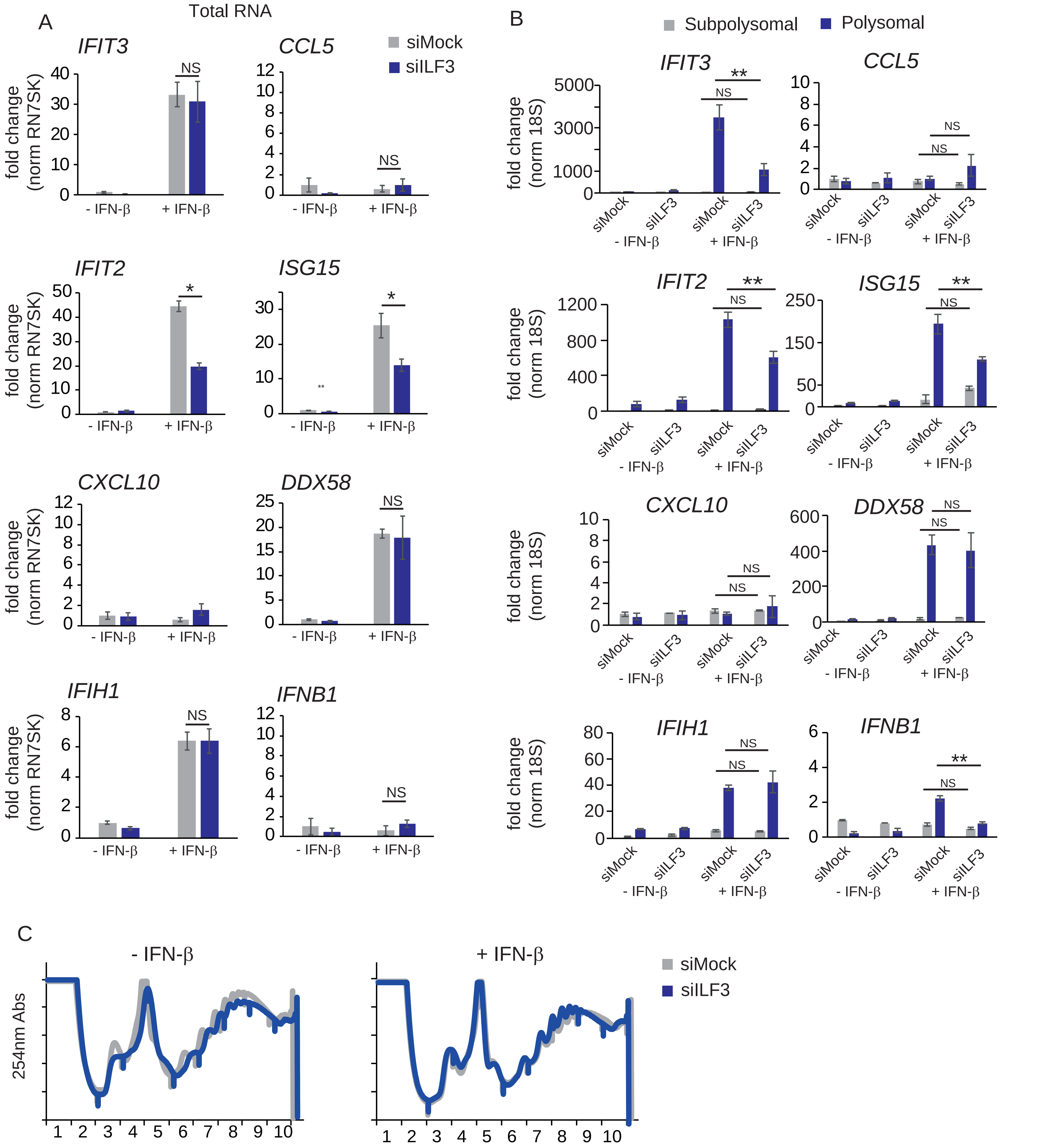

(A) qRT-PCR analyses of ISG and *IFNB1* expression upon 4-hour of exogenous IFN-β stimulation in HeLa cells depleted of ILF3 (siILF3, blue), a non-targeting scramble siRNA was used as a control (siMock, grey). Data show the average (n=3) +/- sem relative to (siMock -IFN-β) and normalized to RN7SK. (*) p-val<0.05 by two-way ANOVA followed by Tukey's multiple comparison test, N.S non-significant

(B) qRT-PCR quantification of ISGs enrichment in subpolysomal (grey) and polysomal (blue) pooled fractions with or without exogenous IFN-β stimulation, in the presence (siMock) or absence of ILF3 (siILF3). Data show the average (n=3)+/- sem normalised to 18S rRNA and relative to subpolysomal level in mock (*) p-val<0.05, (**) p-val<0.001 by two-way ANOVA followed by Tukey's multiple comparison test , n.s. non significant

(C) Sucrose fractionation of cytoplasmic extracts from non (-IFN-β) or stimulated with exogenous IFN-β (+IFN-β) in HeLa cells transfected with scramble siRNAs (siMock) or siRNAs targeting ILF3 (siILF3). UV absorbance, 254nm, is represented in the y-axis for each of the fractions collected after centrifugation (x-axis).

Supplementary Table 1. Oligonucleotides used in this study

Primer Name	Sequence
ACTB F	ACCGAGCGCGGCTACAG
ACTB R	CTTAATGTCACGCACGATTTCC
CCL5 F	GCTGTCATCCTCATTGCTACTG
CCL5 R	AGAGTTGATGTACTCCCGAACC
CXCL10 F	TGACTCTAAGTGGCATTCAAGG
CXCL10 R	CCTTTCCTTGCTAACTGCTTTC
FOS F	AGGAGGGAGCTGACTGATACAC
FOS R	ACAGACATCTCTTCTGGGAAGC
GA17/ EIF3M F	TGCTGCTTCAAAAGTCATGG
GA17/ EIF3M R	AAGGTGGTCAAAAAGAAATGC
IFIH1 F	GGCACCATGGGAAGTGATT
IFIH1 R	ATTTGGTAAGGCCTGAGCTG
IFIT2 F	TTCATAAGATGCGTGAAGAAGG
IFIT2 R	TGCGAAGAACATCTGTTACACC
IFIT3 F	CTGATGCTGAAAAGCAACAATC
IFIT3 R	AACCCTCTAAACCATGTTGCAC
IFNB1 F qPCR	CCTGAAGGCCAAGGAGTACA
IFNB1 R qPCR	AGCAATTGTCCAGTCCCAGA
IFNB1 F semiQ-PCR	CTCTCCTGTTGTGCTTCTCC
IFNB1 R semiQ-PCR	GTCAAAGTTCATCCTGTCCTTG
IL11 F	ACAGCTGAGGGACAAATTCC
IL11 R	AGCTGTAGAGCTCCCAGTGC
IL6 F	CCAGGAGAAGATTCCAAAGATG
IL6 R	GATGATTTTCACCAGGCAAGTC
IL8 F	AGCACACAAGCTTCTAGGACAAG
IL8 R	GGTGGAAAGGTTTGGAGTATGTC
ISG15 F	TGGTGGACAAATGCGACGAA
ISG15 R	CAGGCGCAGATTCATGAAC
NF110 F	CCAAAACACGCTGGGAAGAA
NF110 R	CCATAGTTGCTGTAGGGGCT
NF90 F	ACGGCTATCATGATTTTGGGTC
NF90 R	ACGGACACTTTGGGTTTTGG
EIF2AK2 F	CTGTTGATGGCACTCTGGAA
EIF2AK2 R	GGTCAATTGTGGGCTTCACT
DDX58 F	TGTGGGCAATGTCATCAAAA
DDX58 R	GAAGCACTTGCTACCTCTTGC
TNF F	CTCTCTCTAATCAGCCCTCTGG
TNF R	GTTGACCTTGGTCTGGTAGGAG
RSAD2 F	AAGACCGGGGAGAATACCTG
RSAD2 R	ATCAGGCTTCCATTGCTCAC
18S rRNA F	GATGGTAGTCGCCGTGCC
18S rRNA R	GCCTGCTGCCTTCCTTGG
RNA7SK F	CATCCCCGATAGAGGAGGAC
RNA7SK R	GCGCAGCTACTCGTATACCC
